# Dynamically expressed microRNA-15b modulates the activities of CD8^+^ T lymphocytes in mice with Lewis lung carcinoma

**DOI:** 10.1186/1479-5876-11-71

**Published:** 2013-03-21

**Authors:** Guocheng Zhong, Xiaoming Cheng, Haixia Long, Luhang He, Wei Qi, Tong Xiang, Zhongquan Zhao, Bo Zhu

**Affiliations:** 1 Institute of Cancer, Xinqiao Hospital, Third Military Medical University, Chongqing 400037, People’s Republic of China; 2 Institute of Respiratory Diseases, Xinqiao Hospital, Third Military Medical University, Chongqing 400037, People’s Republic of China

**Keywords:** microRNA-15b, CD8+ T lymphocyte, Immune function, Memory T cell

## Abstract

**Background:**

CD8+ T cells are key members of adaptive immunity against tumorigenesis. As subset of CD8+ T cells, effector T cells (Te) and memory T cells (Tm) have different biological activities. The former can kill tumor cells but come into apoptosis in a certain period and the latter is static with the ability of self-renewal. Previous studies showed that microRNAs (miRNA) played critical roles in regulating adaptive immunity. This study aimed to identify the different expression of miRNAs between Te and Tm cells in tumor-bearing mice and to sort out the target miRNAs which can be regulated to improve anti-tumor activities of CD8+ T cells.

**Methods:**

miRNA expression profiling was performed on CD8+ Te and Tm cells from mice with Lewis lung carcinoma. Differentially expressed miRNA (miRNA-15b) was chosen and analyzed by qRT-PCR. Then, flow cytometry, ELISA, and CFSE kit were used to evaluate the biological effects of miRNA-15b on apoptosis, cytokine secretion, phenotype, and proliferation of CD8+ T cell. The possible downstream target genes of this miRNA were also analyzed.

**Results:**

Analysis of miRNA microarray and qRT-PCR showed that the level of miRNA-15b was higher in CD8+ Tm cells than in Te cells. Higher expression of miRNA-15b was observed in CD8+ T cells from tumor-bearing mice than those from healthy ones. Transfection of CD8+ T cells with miRNA-15b mimics could prevent T cells from apoptosis by inhibiting the translation of DEDD (Death Effector Domain-containing DNA binding protein). Moreover, ectopic miRNA-15b could inhibit the activation of CD8+ T cells (via repressing the production of IL-2 and IFN-γ and expression of CD69) and promote expression of CD44 through unknown pathways.

**Conclusion:**

Up-regulation of miRNA-15b in tumor environment might negatively regulate anti-tumor immunity through inhibiting function of CD8+ T cells. miRNA-15b might be a potential therapeutic target for immunotherapy.

## Background

Cancer is a lethal disease with severe functional deterioration and high mortality. Currently, T cells, particularly CD8+ T cells have been widely employed as effector cells in anti-tumor immunotherapy [1,2]. However, the clinical efficacy of T cell immunotherapy is very limited because of the short lifetime of effector T cells due to their susceptibility to apoptosis in vivo. In addition, anti-tumor activities of T cells are frequently impaired by powerful tumor microenvironments [3]. Therefore, it has attracted many researchers to explore the biological activity of T cells.

During a special cellular immune response, naive T cells activated by antigen would differentiate to effector T (Te) cells with various immune functions. As antigen is cleared, over 95% of Te cells becomes self-apoptotic, and less than 5% of Te cells sequentially differentiates to memory T (Tm) cells which can survive and self-renew in lymphoid tissue for a long term [4]. Is there any regulatory difference which exists between Te and Tm cells? Are there any potential target molecules that can enhance anti-tumor ability of T cells [5]?

Numerous aspects involving including antigenic stimulation signal, T cell microenvironment, cytokine productions, and T cell surface molecules [6-8] have been investigated. More recently, microRNAs (miRNA), which participate in immunological regulation, have attracted much attention [9]. miRNAs are non-coding small molecule RNA of about 22 nucleotides and can degrade target mRNAs or inhibit their translation. Cellular proliferation, differentiation, growth, metabolism and many other biological processes are influenced by miRNAs [10,11]. Substantial studies have shown that the miRNAs are involved in subtle or accurate regulation of T cells [12,13]. So far, related researches mainly concentrate on the miRNAs that regulate CD4+ T cells, such as miRNA-146a [14], miRNA-155 [15], miRNA-17~92 [16]. Very few miRNAs that regulate CD8+ T cells have been reported. In this study, we performed the miRNA microarray analysis on differential expression between CD8+ Te and Tm cells from mice with Lewis lung carcinoma (Additional file 1: Table S1) and identified the target miRNAs differentially expressing in T cell subsets (Te and Tm cells). Among the miRNAs whose expression in Tm was higher than in Te, the level of miRNA-15b was higher in CD8+ T cells from tumor-burdened mice than those from healthy mice. The effects of miRNA-15b on apoptosis, phenotype, cytokine secretion and proliferation of CD8+ T cells were studied and the possible target genes of miRNA-15b also analyzed.

## Materials and methods

### Establishment of tumor-burdened mice model

The Lewis lung carcinoma cell line was purchased from ATCC (American Type Culture Collection). Female C57BL/6 mice (4–6 weeks old) were obtained from the Chinese Academy of Medical Sciences (Beijing, China). All mice were fed with irradiated food and were executed by cervical dislocation. Lewis lung carcinoma cells in growth logarithm period were re-suspended in phosphate-buffered saline (PBS). 1 × 10^5^ cells were injected into the armpit and groin regions of C57 BL/6 mice respectively. These tumor-burdened mice were continually raised to 4–5 weeks until the tumors grew up to about 3 × 3 cm (Additional file 2: Figure S1). The ethical approval of animal experiment was authorized by Third Military Medical University and all mice Animals were handled following an Institutional Animal Care and Use Committee-approved protocol.

### Isolation of effector CD8+ Te cells and CD8+ Tm cells from tumor-burdened mice

4–5 weeks after injection of Lewis lung carcinoma cells into C57 BL/6, CD8+ Te and CD8+ Tm cells were isolated, purified and pooled from the spleens of 10 tumor-burdened mice by fluorescence-based cell sorting [17]. The purity of Tm (CD8^+^CD44^+^CD197^+^) and Te (CD8^+^CD44^-^CD69^+^) was more than 90%. All fluorescence antibodies (CD8-FITC/CD44-PE/CD69-APC/CD197-APC monoclonal antibodies) were purchased from BD Pharmingen™ or BioLegend.

### Cell culture, co-stimulation and activation

CD8+ T cells from spleens of tumor-burdened mice or healthy ones were isolated by positive selection with anti-CD8 magnetic beads (Order No. 130-049-401, Miltenyi Biotec) and were adjusted to 1 × 10^6^ cells/ml in RPMI(Roswell Park Memorial Institute)-1640 supplemented with L-glutamine, penicillin-streptomycin, sodium pyruvate (Hyclone), nonessential amino acids (Invitrogen) and 10% FCS (Hyclone). For activation of CD8+ T cells, monoclonal anti-CD3 antibody (CD3 mAb) at a final concentration of 2.5 μg/mL (145-2C11, eBiosicence) was placed into 96-well plates and incubated at 4°C for 8-10 h. Then, the medium of CD3 mAb was removed carefully and the plates were washed twice with PBS followed by placing CD8+ T cells into the plates (100μL cells per well). Then, the CD8+ T cells were co-stimulated with CD28 mAb at a final concentration of 2 μg/mL (35.71, eBiosicence) in combination with CD3 mAb for 24 to 120 h at 37°C and 5% CO_2_[18].

### RNA extraction, miRNA microarray and quantitative real-time PCR

Total RNA of CD8+ Te or Tm cells were prepared with TRIzol (Invitrogen) and extracted for miRNA microarray analysis or quantitative real-time PCR assay (total RNA of CD8+ T cells from tumor-burdened mice or healthy mice were prepared similarly). miRNA microarray experiments were performed using Affymetrix Genechip miRNA 2.0 Array (miRNA QC tool version 1.1.1 or “AffyMir” version 1.1.1 was used for data analysis). For miRNA quantitative reverse-transcribed PCR (qRT-PCR), extracted total RNA was reverse-transcribed with specific primer of miRNA-15b (RIBOBIO, Guangzhou, China) by using the PrimeScript^R^ RT reagent Kit with gDNA Eraser (TaKaRa, Japan) on an ABI PRISM 7500 Real-Time PCR system (Applied Biosciences, Benicia, CA, USA). qRT-PCR of miRNA-15b was performed at the following conditions: 95°C 30 s, followed by 40 cycles at 95°C for 5 s, 60°C for 34 s and 70°C for 45 s. U6 gene was used as a normalization control.

### Transfection of miRNA-mimics

Fresh CD8^+^ T cells (1 × 10^6^/mL) from spleen of tumor-burdened mice were transfected with 50nM miRNA-15b mimics (mimic-15b) or negative-control scrambled miRNA mimics (mimic-NC, RIBOBIO) using Lipofectamine™ 2000 (11668–027, Invitrogen) in reduced serum (Opti-MEM, 31085–062, Invitrogen) following the manufacturer’s protocol [19]. The transfection rate of T cells was measured by flow cytometry or qRT-PCR 12 h later.

### Assay of apoptosis

CD8^+^ T cells transfected with mimic-15b or mimic-NC (5 × 10^5^ cells/mL) were grown in RPMI-1640. The apoptosis of these T cells were induced by CD3 mAb (5 μg/mL, clone 145-2C11) for 12 h. Apoptotic cell fractions were identified by double staining of annexin V–fluorescein isothiocyanate (FITC) and propidium iodide (PI) using an apoptosis detection kit (K101-25, BioVision) according to the manufacturer’s instructions. Apoptotic cells were analyzed by flow cytometry.

### ELISA

CD8+ T cells from spleens of tumor-burdened mice were transfected with mimic-15b or mimic-NC and were co-activated by CD3/CD28 mAb. The culture supernatant was then collected, and the IL-2 and IFN-γ in supernatant were detected with ELISA kits (EK0398, EK0375, Boster) according to the manufacturer’s instructions [20]. Plates were read at an absorbance of 450 nm (A450) using a Sunrise microplate reader. IL-2 and IFN-γ were tested in quadruplicates in three independent ELISA measurements.

### Flow cytometry

Transfected CD8^+^ T cells with mimic-15b or mimic-NC were co-activated respectively, as described above. Before and 48–120 h after activation, The transfected CD8^+^ T cells were stained with APC-labeled CD69 mAb, PE-labeled CD44 mAb or amouse isotype control (BD Pharmingen™) at 4°C for 30 min and the expression of CD69 or CD44 in CD8^+^ T cells was determined by flow cytometry.

### Cell proliferation assay

To detect proliferation rate, CD8+ T cells transfected with mimic-15b or mimic-NC were labeled with 0.6 mM Carboxyfluorescein Diacetate (CFSE) dye (CellTrace™ CFSE Cell Proliferation Kit, C34554, Invitrogen) for 10 min at 37°C and the staining was ended by adding 5 volumes of ice-cold culture media to the cells. Labeled CD8+ T cells were co-activated for 96 h. Cell proliferation was then assessed by flow cytometry and analyzed by FlowJo software version 6.3 [21].

### Luciferase reporter experiments

To confirm target genes of miRNA-15b, we performed luciferase reporter assays on DNA constructs (based on pGL-3-luc vector, Promega) containing the firefly luciferase gene, which was linked to the 3-untranslated region (UTR) of each potential target including wild type or mutants (bases 2–6 in the seed region where miRNA-15b bind to target gene’ 3-UTR were mutated). The reporter constructs were transiently co-transfected in 293T cells with mimic-15b or mimic-NC, then, the luciferase activity was measured using the Dual-Luciferase assay kit (Promega) according to manufacturer’s instructions. Renilla luciferase reporter was used to normalize firefly luciferase readings.

### Detection of target gene by qRT-PCR and Western blot

mRNA of DEDD and a normalization control of β-actin were measured by qRT-PCR using the special primers: DDED-For: 5^′^-AGCTGCTGGTGAACGTGGATGAG-3^′^; DDED-Rev: 5^′^-TCAGGGTAATGCCTGCAGCATCAAG-3^′^; β-actin-For: 5^′^-TCAAGATCATTGCTCCTCCTGAG-3^′^; β-actin-Rev: 5^′^-ACATCTGCTGGAAGGTGGACA-3^′^ (RIBOBIO). RNA extraction and qRT-PCR were performed as mentioned above. PCR were carried out for 35 cycles at 94°C for 30 s, 69°C for 30 s and 74°C for 60 s. Relative expression level was calculated with Comparative Ct method.

For Western blots, a specific mAb of DEDD (G6) was used (sc-271191, Santa cruz biotechnology). Mouse mAb β-actin (C4) was used to detect β-actin as loading control (sc-47778, Santa cruz biotechnology). CD8+ T cells were lysed, and extracted proteins were separated with polyacrylamide gel electrophoresis. After that, proteins were transferred onto a polyvinylidene difluoride membrane (Millipore). Membranes were washed with Tris buffered saline Tween (TBST), blocked in 5% non-fat dry milk [22], and blotted with DEDD (1:250) or β-actin (1:1,000) antibodies. The image was quantified using Image J software version 10.2.

### Statistical analysis

All data were analyzed by SAS (Statistical Analysis System) software. The statistical significance of differences was determined with student *t*-test for pairwise comparison. The difference between 2 groups was considered significant when *P* < 0.05.

## Results

### miRNA-15b was upregulated in CD8+ Tm from tumor-burdened mice

**Figure 1:**
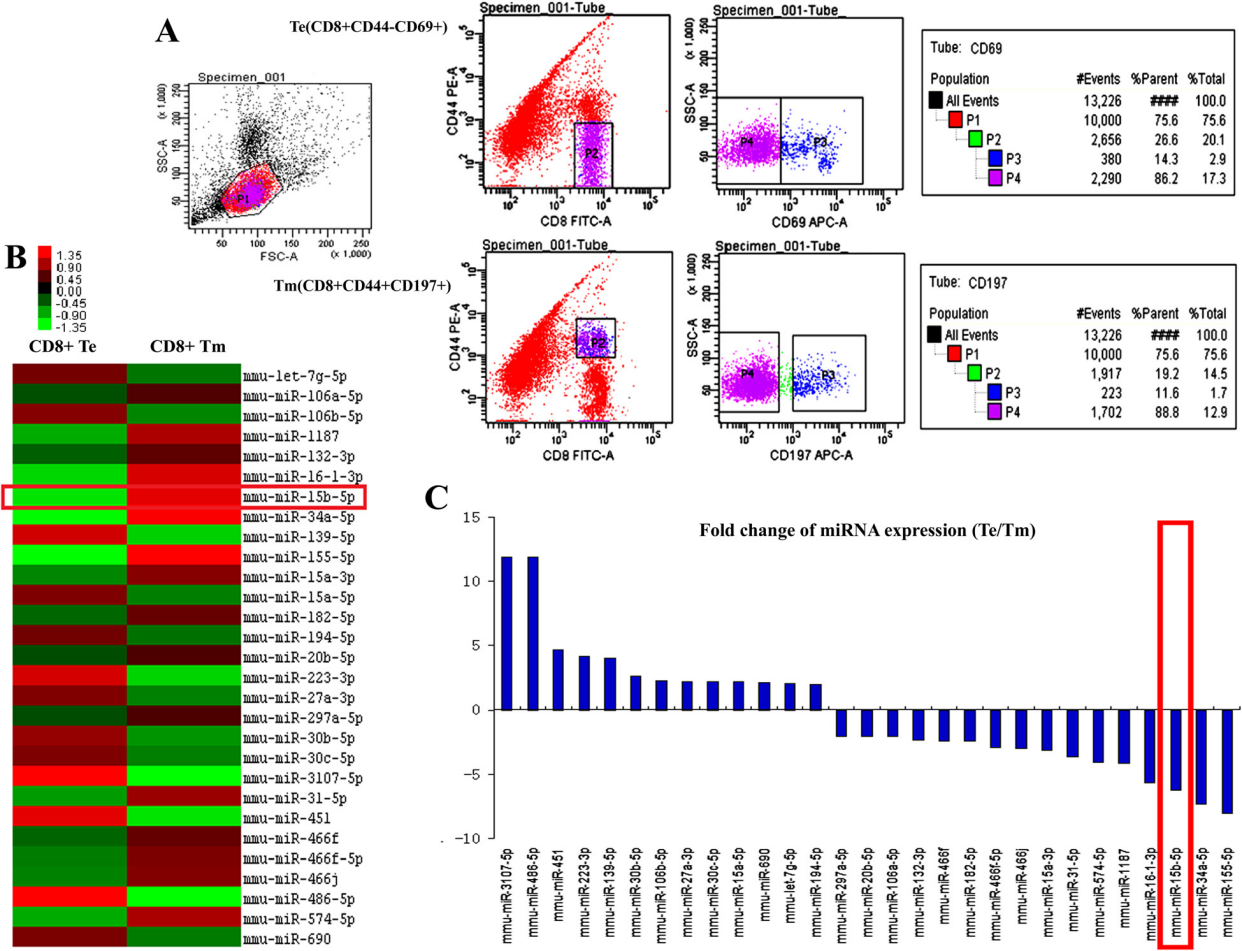
**The expression of miRNA-15b between CD8+ effector T (Te) cells and memory T (Tm) cells from the spleens of tumor-burdened mice. A)** CD8+ Te cells labeled with CD8 + (FITC)CD44-(PE)CD69 + (APC) and CD8+ Tm cells labeled with CD8 + (FITC)CD44 + (PE)CD197 + (APC) were purified by fluorescence-based cell sorting. **B)** Diagram shows the differentially expressed miRNAs between CD8+ Te and Tm cells. Each row represents a single miRNA. Red color represents that the miRNA was highly expressed and green color represents low expression level. **C)** Main miRNAs which were differentially expressed in CD8+ Te and Tm cells were listed. 9 of them were down-regulated and 15 of them were up-regulated in Tm cells.

**Figure 2:**
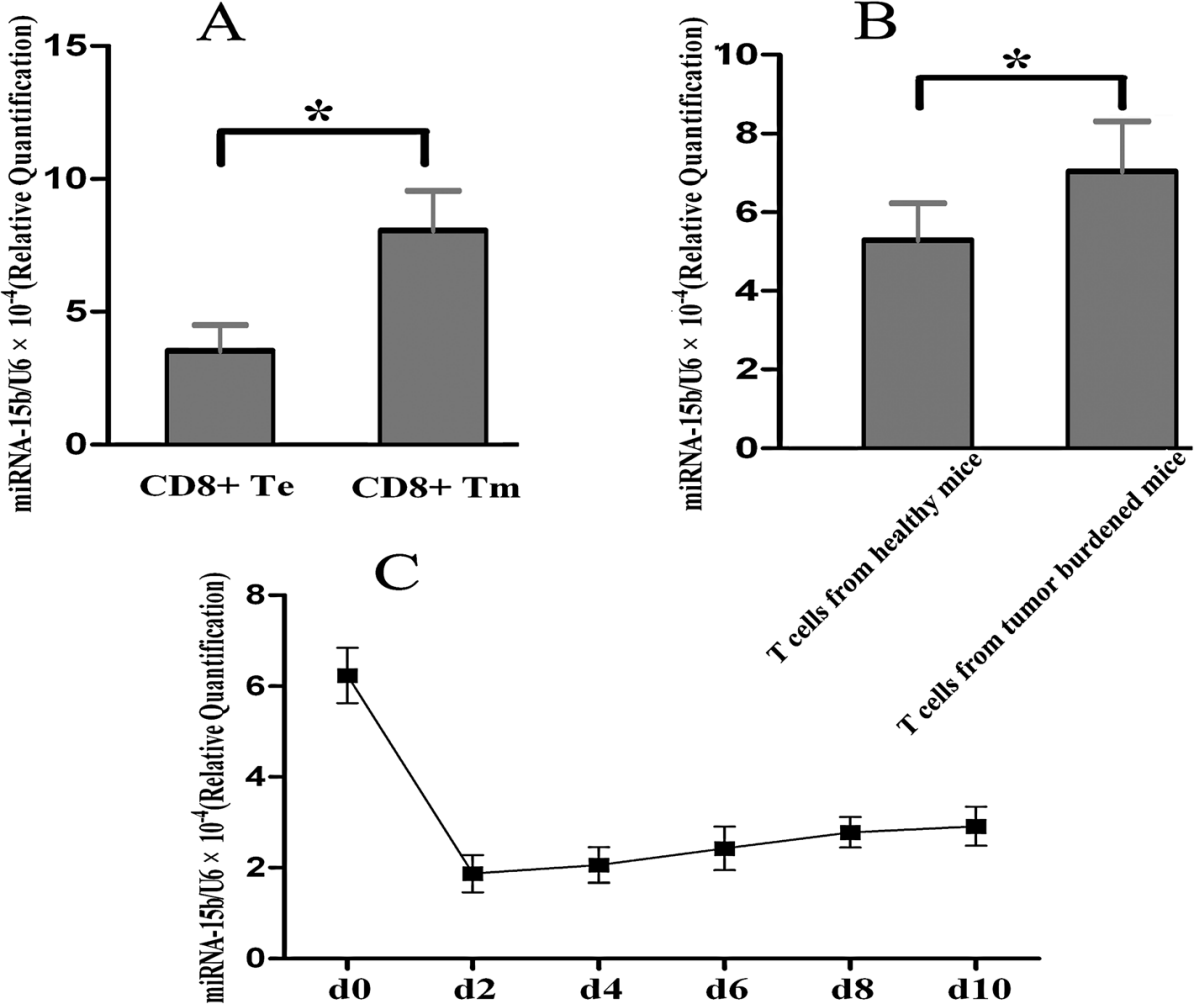
**Dynamic expression of miRNA-15b in CD8+ T cells was detected by qRT-PCR. A)** The relative expression of miRNA-15b in CD8+ Te and Tm cells was detected by qRT-PCR. U6 was used as an internal control to normalize the relative amounts of miRNA-15b. *P < 0.01. **B)** Relative expression of miRNA-15b was detected in CD8+ T cells from tumor-burdened mice or healthy ones by qRT-PCR. The results are presented as mean ± standard deviation and obtained from 6 independent experiments (6 tumor-burdened mice vs 6 healthy mice). *P < 0.05. **C)** CD8+ T Cells were co-stimulated by CD3/CD28 mAb and the levels of miRNA-15b were measured at different time points by qRT-PCR.

Results from the miRNA microarray displayed that 25 miRNAs were differentially expressed in CD8+ Tm and Te cells (Figure 1A). 9 miRNAs were down-regulated and 15 miRNAs were up-regulated in Tm cells, compared with Te cells (Table 1). As shown in Figures 1B and C, miRNA-15b was one of the miRNAs that had higher levels in CD8+ Tm, consistent with a previous study [23]. The data were confirmed by qRT-PCR (Figure 2A). Further analysis indicated that the level of miRNA-15b in CD8+ T cells from the spleens of tumor-burdened mice was also higher than those from healthy ones (Figure 2B). The role of miRNA-15b in regulating function of CD8+ T cells is not reported previously. We then chose it for further investigation.

**Table 1:** The list of differentially expressed microRNAs between CD8+ Te and Tm cells

**ProbeSet Name**	**Mature1_Acc**	**mirbaseV18**	**CD8**^**+**^**Te**	**CD8**^**+**^**Tm**	**Style**	**Log2**
mmu-miR-486_st	MIMAT0003130	mmu-miR-486-5p	8.906548	5.336512	up	3.570036
mmu-miR-139-5p_st	MIMAT0000656	mmu-miR-139-5p	7.818672	5.802054	up	2.016618
mmu-miR-30b_st	MIMAT0000130	mmu-miR-30b-5p	7.390241	6.001204	up	1.389037
mmu-miR-106b_st	MIMAT0000386	mmu-miR-106b-5p	9.228545	8.014892	up	1.213653
mmu-miR-27a_st	MIMAT0000537	mmu-miR-27a-3p	8.051024	6.890368	up	1.160656
mmu-miR-30c_st	MIMAT0000514	mmu-miR-30c-5p	8.109971	6.963645	up	1.146326
mmu-miR-15a_st	MIMAT0000526	mmu-miR-15a-5p	6.800948	5.663513	up	1.137387
mmu-miR-690_st	MIMAT0003469	mmu-miR-690	8.572385	7.465322	up	1.107063
mmu-miR-194_st	MIMAT0000224	mmu-miR-194-5p	6.475734	5.470155	up	1.005579
mmu-miR-20b_st	MIMAT0003187	mmu-miR-20b-5p	8.066008	9.086855	down	−1.020847
mmu-miR-106a_st	MIMAT0000385	mmu-miR-106a-5p	8.328273	9.380265	down	−1.051992
mmu-miR-132_st	MIMAT0000144	mmu-miR-132-3p	5.455552	6.673903	down	−1.218351
mmu-miR-466f_st	MIMAT0005844	mmu-miR-466f	5.642788	6.931221	down	−1.288433
mmu-miR-182_st	MIMAT0000211	mmu-miR-182-5p	4.408060	5.698591	down	−1.290531
mmu-miR-466f-5p_st	MIMAT0004881	mmu-miR-466f-5p	5.711618	7.241209	down	−1.529591
mmu-miR-466j_st	MIMAT0005848	mmu-miR-466j	5.609854	7.204075	down	−1.594221
mmu-miR-15a-star_st	MIMAT0004624	mmu-miR-15a-3p	4.456201	6.089243	down	−1.633042
mmu-miR-31_st	MIMAT0000538	mmu-miR-31-5p	4.899204	6.755762	down	−1.856558
mmu-miR-574-5p_st	MIMAT0004893	mmu-miR-574-5p	5.894817	7.919928	down	−2.025111
mmu-miR-1187_st	MIMAT0005837	mmu-miR-1187	5.847568	7.887634	down	−2.040066
mmu-miR-16-star_st	MIMAT0004625	mmu-miR-16-1-3p	5.524515	8.019949	down	−2.495434
mmu-miR-15b_st	MIMAT0000124	mmu-miR-15b-5p	6.722845	9.358693	down	−2.630109
mmu-miR-34a_st	MIMAT0000542	mmu-miR-34a-5p	4.728513	7.604746	down	−2.876236
mmu-miR-155_st	MIMAT0000165	mmu-miR-155-5p	7.589617	10.60282	down	−3.013203

### Expression of miRNA-15b was altered during T cell activation and differentiation

To understand the roles of miRNA-15b in CD8+ T cell activation and differentiation, we treated CD8+ T cells with monoclonal antibodies against CD3 and CD28. As shown in Figure 2C, the level of miRNA-15b was significantly upregulated in non-activated CD8+ T cells and progressively decreased upon CD3/CD28 co-stimulation. After 48 h, the expression of miRNA-15b was re-elevated slowly.

### Transfection efficiency of CD8+ T cells

To investigate how miRNA-15b regulates functions of CD8+ T cells, we synthesized miRNA-15b mimics and labeled them with cy3 (mimic-15b-cy3). Then CD8+ T cells were transfected with mimic-15b-cy3 [24]. Efficiency of transfection was determined by flow cytometric analysis of positive cells with red fluorescence (cy3 display red fluorescence). The data showed that about 45~55% CD8+ T cells were positive. qRT-PCR analysis confirmed that miRNA-15b mimics were successfully transfected into CD8+ T cells (Additional file 3: Figure S2).

### MiRNA-15b repressed CD8+ T cell apoptosis induced by activation-induced cell death (AICD)

**Figure 3:**
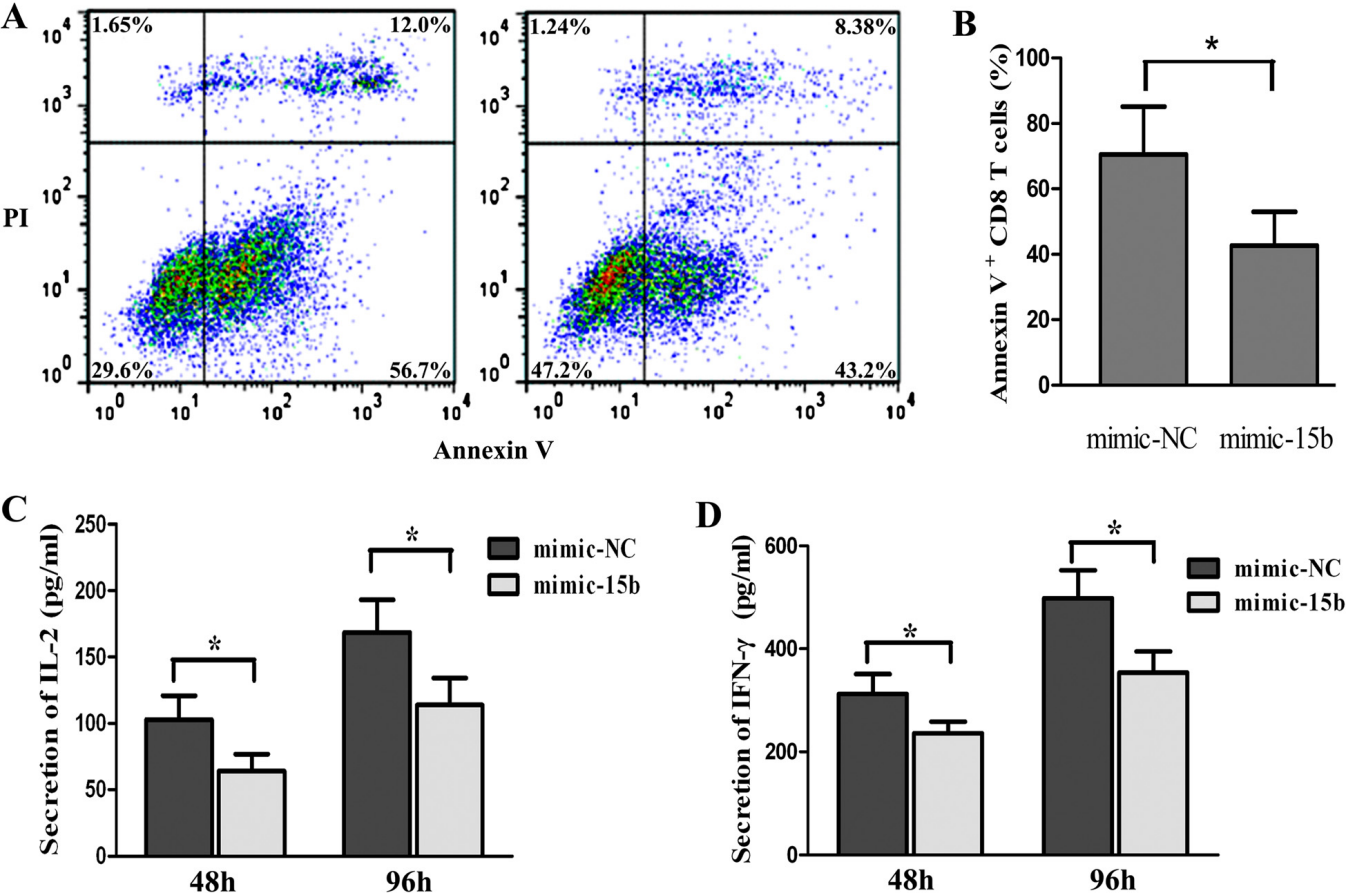
**Ectopic expression of miRNA-15b protects CD8+ T cells from apoptosis and impedes the production of IL-2 and IFN-γ. A)** 12 h after adding the anti-CD3 antibody, flow cytometry showed that CD8+ T cells with mimic-15b (right) displayed less apoptosis than T cells with mimic-NC (left). **B)** Apoptotic CD8+ T cells (which were labeled as annexin V-FITC positive) were evaluated. The result was derived from three independent assays (mean ± standard deviation). *P < 0.01. **C)** CD8+ T cells transfected with mimic-15b or mimic-NC were co-stimulated with CD3/CD28 mAb for 24 h and 48 h, IL-2 levels were determined by ELISA assay. Each value is the mean of 3 independent detections. *P < 0.01. **D)** 24 h and 48 h after co-stimulation, IFN-γ levels were determined by ELISA assay. Each value was the mean of 3 independent detections. *P < 0.01.

Repetitive stimulation of TCR on T cells can lead to apoptosis of activated T cells. This process is mainly mediated by AICD and FAS/FAS ligand system. This apoptosis can be induced in vitro by ligation of TCR complex with the anti-CD3 antibody [25]. Our data showed that the apoptosis in CD8+ T cells transfected with mimic-15b was less than that in control T cells transfected with mimic-NC at 12 h after adding the anti-CD3 antibody, (Figures 3A and B), indicating that miRNA-15b may be a negative regulator of AICD.

### MiRNA-15b inhibited the production of IL-2 and IFN-γ in CD8+ T cells

To investigate whether miRNA-15b regulates the secretion of IL-2 and IFN-γ, which are important for anti-tumor immunity, CD8+ T cells transfected with mimic-15b or mimic-NC were co-activated by the antibodies against CD3 and CD28. Supernatants from the transfected T cells were collected at 24 h and 48 h after co-activation and production of IL-2 and IFN-γ was measured by ELISA kit. As shown in Figures 3C and D, the secretion of IL-2 and IFN-γ was significantly reduced in the T cells transfected with mimic-15b upon co-stimulation, compared with the T cells transfected with mimic-NC (*P* < 0.05). The results suggest that miRNA-15b may impair the anti-tumor activities of CD8+ T cells [26].

### MiRNA-15b regulated phenotype of CD8+ T cells but did not affect proliferation of T cells

**Figure 4:**
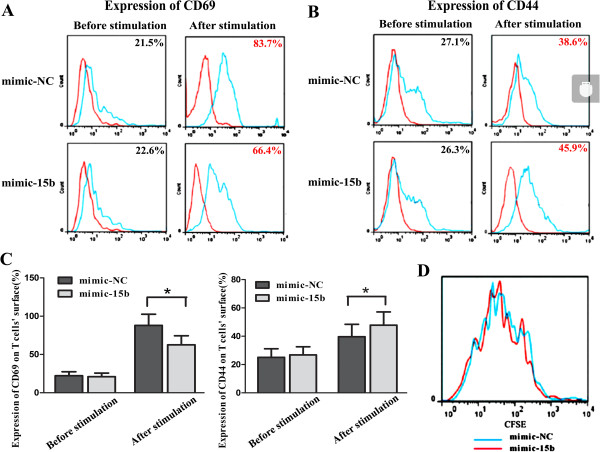
**miRNA-15b regulates phenotypes of CD8+ T cell but does not affect proliferation of T cell. A)** After transfected with mimic-15b or mimic-NC, CD8+ T cells were co-stimulated by CD3/CD28 mAb. The expression of CD69 before or 48 h after co-stimulation was detected by flow cytometry analysis. The red line represented an isotype-matched control. The blue line represented the staining of CD69 mAb conjugated with APC. **B)** The expression of CD44 before or 120 h after co-stimulation was detected by flow cytometry analysis. The red line represented an isotype-matched control. The blue line represented the staining of CD44 mAb conjugated with PE. **C)** Before and after co-stimulation by CD3/CD28 mAb, the positive rates of CD69 (left) or CD44 (right) in CD8+ T cells with mimic-15b or mimic-NC were evaluated. The data were obtained from three independent experiments (mean ± standard deviation), *P < 0.01. **D)** CFSE-dilution analysis on CD8+ T cells transfected with mimic-15b or mimic-NC after 96 h of co-stimulation with CD3/CD28 mAb. Red line represented T cells with mimic-15b and blue line represented T cells with mimic-NC.

CD69 is a marker of activation of T cell and CD44 is definitive indicator of memory cells. To investigate whether miRNA-15b influences the phenotypes of T cells, the levels of CD69 and CD44 in CD8+ T cells transfected with mimic-15b or mimic-NC were measured after co-stimulation [27]. The results showed that the expression of CD69 in CD8+ T cells transfected with mimic-15b was lower than that in control T cells transfected with mimic-NC, while the expression of CD44 was higher in T cells transfected with mimic-15b than in control T cells (Figures 4A, B and C).

The effect of miRNA-15b on the proliferation of CD8+ T cells was determined at 96 h after co-stimulation with the antibody against CD3 and CD28 using CFSE kit. Figure 4D shows that there was no significant difference between CD8+ T cells transfected with mimic-15b and control T cells (*p* > 0.05).

### Prediction and validation of target genes of miRNA-15b

**Figure 5:**
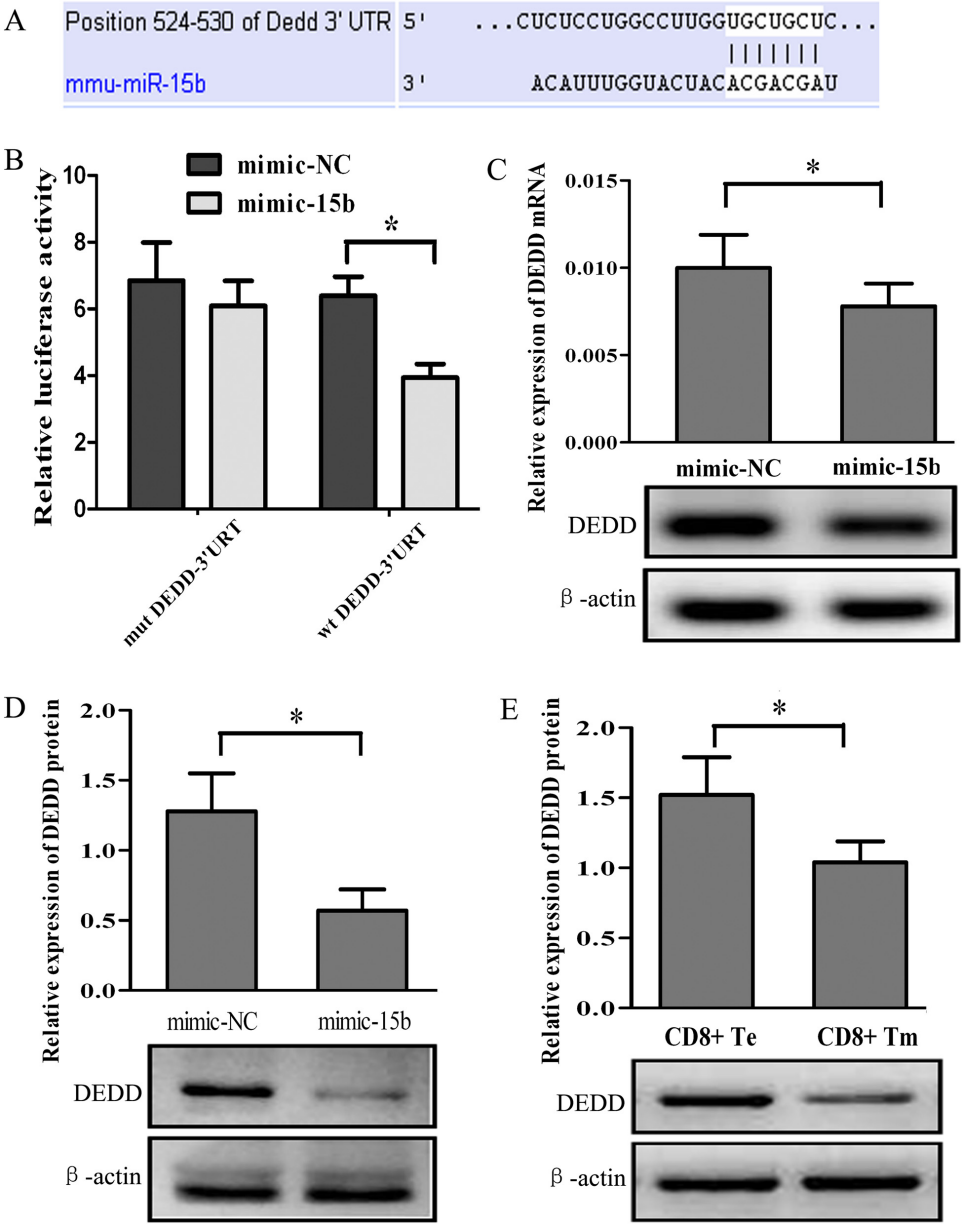
**Prediction and validation of DEDD as target gene of miRNA-15b. A)** Schematic representation of miRNA-15b’s sequence bound to DEDD. **B)** The luciferase constructs fused to 3^′^UTR (or mut) of DEDD were co-transfected in 293 T cells with mimic-15b or mimic-NC. The luciferase activities were expressed as mean ± standard deviation (n = 3) of the ratio of firefly luciferase over renilla luciferase (*P < 0.01). **C)** CD8+ T cells were isolated from C57BL/6 mice and transfected with mimic-NC or mimic-15b, the levels of DEDD mRNA in T cells with mimic-NC or mimic-15b were measured by qRT-PCR or PCR electrophoresis (72 h after transfection, * *P* < 0.05). **D)** The levels of DEDD protein in CD8+ T cells with mimic-15b or mimic-NC were assessed by western blot (72 h after transfection, * *P* < 0.01). **E)** CD8+ Te and Tm were prepared from the spleens of C57BL/6 mice with Lewis lung carcinoma. The levels of DEDD protein in CD8+ Te or Tm were assessed by western blot (* *P* < 0.05).

To reveal the biological relevance of miRNA-15b to the functions of CD8+ T cells, such as activation, apoptosis, or proliferation, we searched for the potential targets of miRNA-15b by using bioinformatics tools (miRGen, TargetScan and PicTar). The result showed that about 600 genes were predicted as the targets of miRNA-15b. We selected 8 putative target genes, which are involved in activities of T cells (Table 2 and Figure 5A). We constructed the luciferase reporter constructs containing the 3-untranslated region (UTR) of each potential target genes, including CD28, TNF receptor-associated factor 3, DEDD, interleukin-1 receptor-associated kinase 2, E2F transcription factor 3, programmed cell death 6 interacting protein, lipopolysaccharide-induced TNF factor and Interleukin-15. As demonstrated by luciferase reporter assays, the transfection with mimic-15b significantly reduced the luciferase activities of the reporter gene fused to the DEDD 3^′^UTR, but other potential targets were not affected by mimic-15b overexpression. Mutation of 4 nucleotides in the 3^′^UTR seed sequence of DEDD could reverse the inhibitory effect of mimic-15b, indicating that the effect was specific (Figure 5B). To further confirm that DEDD is regulated by miRNA-15b, we transfected mice CD8+ T cells with mimic-15b or mimic-NC. We observed that at 72 h after transfection, the levels of DEDD mRNA and protein in T cells transfected with mimic-15b were significantly decreased (Figures 5C and D). In addition, the protein level of DEDD in Tm cells from tumor-burdened mice was lower than that in Te cells (Figure 5E). There is no difference of the level of DEDD mRNA between Tm and Te cells (*p* > 0.05, data not shown).

**Table 2:** The list of predicted target genes of miRNA-15b after selection

**Target gene**	**Gene name**	**Possible function**	**Sites**	
			**Conserved**	**Poorly conserved**
CD28	CD28 molecule	activation	1(8mer)	0
TRAF3	TNF receptor-associated factor 3	apoptosis	1(7mer-m8)	3(8mer)
DEDD	death effector domain containing	apoptosis	1(7mer-m8)	2(7mer-m8, 7mer-1A)
IRAK2	interleukin-1 receptor-associated kinase 2	apoptosis	1(8mer)	0
E2F3	E2F transcription factor 3	proliferation	1(8mer)	1(7mer-m8)
PDCD6IP	programmed cell death 6 interacting protein	Cell cycle	1(7mer-m8)	1(7mer-m8)
LITAF	lipopolysaccharide-induced TNF factor	apoptosis	1(7mer-m8)	0
IL-15	Interleukin-15	activation	1(7mer-m8)	0

## Discussion

It has been known that the differentiation and function of T cells are accurately regulated by miRNAs [28,29]. Abnormality of this regulation is linked to immunodeficiency and tumorigenesis [30]. On the other hand, being one of T cell subsets, Te cells which decide the strength and direction of a special anti-tumor immune response have short survival time and Tm cells whose function is relative static could exist over long periods in vivo. In this study, we aimed to identify the miRNAs that influence the functions of CD8+ T cells and investigate the possible mechanisms by which CD8+ T cells are regulated.

In order to predict the potential miRNAs which were involved in regulating differentiation of CD8+ T cells, especially in tumor-burdened environments, we established a tumor-burdened mice model with Lewis lung carcinoma and isolated the CD8+ Te and Tm cells [31]. The analysis of miRNA microarray showed that the expression of miRNA-15b was higher in CD8+ Tm cells than in Te cells [32]. This result was verified by qRT-PCR. We found that the level of miRNA-15b in CD8+ T cells from tumor-burdened mice was also higher than those from healthy ones, indicating that miRNA-15b may be involved in regulating CD8+ T cells and influenced by tumor environment. We noticed that co-stimulation with the anti-CD3 and CD28 antibody remarkably repressed the expression of miRNA-15b within 48 h, but the miRNA-15b level was restored slowly later, suggesting that the fluctuation of the level of miRNA-15b may associate with the activation of CD8+ T cells.

Compared with Te cells, Tm cells have more potent anti-apoptotic ability. As the expression of miRNA-15b was higher in Tm cells than in Te cells, we examined the effect of miRNA-15b on survival of CD8+ T cells. Our results showed that overexpression of miRNA-15b prevented CD8+ T cells from apoptosis induced by the anti-CD3 antibody, suggesting that miRNA-15b may affect the survival of CD8+ T cells in tumor microenvironment.

In addition to apoptosis, we also tested other effects of miRNA-15b on CD8+ T cells. We demonstrated that overexpression of miRNA-15b in CD8+ T cells impaired the production of IL-2 and IFN-γ in CD8+ T cells. Flow cytometry analysis showed that miRNA-15b can inhibit the expression of CD69, an early activation marker of T cells and upregulate the expression of CD44, a memory marker. These findings indicated that miRNA-15b may play a negative role in activation of CD8+ T cell and induce T cells to a memory state (Besides, miRNA-15b did not show any effects on proliferation of T cells). Meanwhile, these data collectively suggest that the regulated mechanism of miRNA-15b is very complicated.

DEDD is a highly conserved and ubiquitous death effector domain containing protein. It is widely expressed in a variety of tissues and induces apoptosis through its N-terminal DED motif. Bioinformatics analysis and the luciferase reporter assays revealed that DEDD is a potential target of miRNA-15b. Transfection with mimic-15b resulted in a significant reduction in the levels of DEDD mRNA and protein in T cells, consistent with the anti-apoptotic effect of miRNA-15b observed in CD8+ T cells [33]. In addition, our data implied that miRNA-15b may block the translation of DEDD rather than induce the degradation of DEDD mRNA, since DEDD mRNA level was not changed in the Te and Tm cells [34].

In summary, miRNA-15b which was highly expressed in CD8+ T cells was differentially expressed between Tm and Te cells in a tumor-burdened environment. It regulated apoptosis of CD8+ T cells by inhibiting the translation of DEDD and repressed CD8+ T cells activation by certain pathways (which have not yet been confirmed or clarified). Increased expression of miRNA-15b in a tumor-burdened environment could improve anti-apoptotic ability of CD8+ T cells, but at the same time, it also greatly compromised anti-tumor effect of T cell (such as production of IFN-γ) and might cause these T cells to lose responses to tumors [35]. That is to say, miRNA-15b which functions in complex molecular networks of tumor environment seems to exert negative effect and be a potential biomarker in special anti-tumor immunity of CD8+ T cells [36]. The present results provided a preliminary exploration to improve T cells immunotherapy by regulating miRNAs, but further studies are still needed to confirm and apply related conclusion.

## Conclusions

miRNA-15b was highly expressed in CD8+ T cells from tumor-burdened mice. Tm cells expressed higher level of miRNA-15b than Te cells. Upregulation of miRNA-15b expression greatly compromise the anti-tumor effect of T cells from the tumor-burdened mice, although the anti-apoptotic abilities of CD8+ T cells were enhanced. Our data suggest that miRNA-15b may play a suppressive role in the activation of CD8+ T cells and it may be a potential target for anti-tumor immunotherapy.

## Abbreviations

miRNA: microRNA; qRT-PCR: quantitative reverse-transcribed polymerase chain reaction; Te: Effector T cells; Tm: Memory T cells; ATCC: American type culture collection; FITC: Fluorescein isothiocyanate; PE: Phycoerythrin; APC: Allophycocyanin; PI: Propidium iodide; ELISA: Enzyme-linked immunosorbnent assay; AICD: Activation-induced cell death; DEDD: Death effector domain-containing DNA binding protein; CFSE: Carboxyfluorescein diacetate; 3’UTR: 3-Untranslated region.

## Competing interests

All authors declare that they have no competing interests.

## Authors’ contributions

GZ participated in the conception of the study, experimental design, performed assays, and was the primary writer of the paper. LH and ZZ was involved in the conception of the study and took a primary role in culturing the differentiated T cells. XC and WQ participated in experimental design, troubleshooting, editing the manuscript and statistical analysis. HL assisted in microRNA array and data analysis, expression studies and analysis. TX and helped with the ELISA and Western blot. BZ conceived the study, mentored primary authors, and heavily participated in experimental design and data analysis. All authors read and approved the final manuscript.

## Supplementary Material

Additional file 1: Table S1 Raw data of miRNA microarray of CD8+ Te and Tm cells from mice with Lewis lung carcinoma. Complete information of differential expression of miRNA in CD8+ Te and Tm cells from tumor-burdened mice was listed in this table. Click here for file

Additional file 2: Figure S1 Lewis lung carcinoma cells and Tumor-burdened mice. A) The Lewis lung carcinoma cells in growth logarithm period. B) Tumor-burdened C57BL/6 mice were raised for 4 weeks until the lump grew up to 3 × 3 cm. Click here for file

Additional file 3: Figure S2 Transfection of mimic-miRNA in CD8+ T cells. A) CD8+ T cells transfected with mimic-NC (left) or mimic-15b-cy3 (right) were imaged by fluorescence microscopy (red sparkle). B) Representative cytometrical plots showed transfection efficiency of mimic in CD8+ T cells. Red line represented that T cells were transfected with mimic-NC, blue line represented that T cells were transfected with mimic-15b-cy3. C) The relative expression of miRNA-15b was determined by qRT-PCR in T cells with mimic-NC or mimic-15b. U6 was used as an internal control to normalize relative amounts of miRNA. Click here for file
