# Supplementary figures and images for: Dynamically expressed microRNA-15b modulates the activities of CD8+ T lymphocytes in mice with Lewis lung carcinoma
Source: J Transl Med. 2013 Mar 21;11:71. doi: 10.1186/1479-5876-11-71 (PMC3608092; doi:10.1186/1479-5876-11-71)

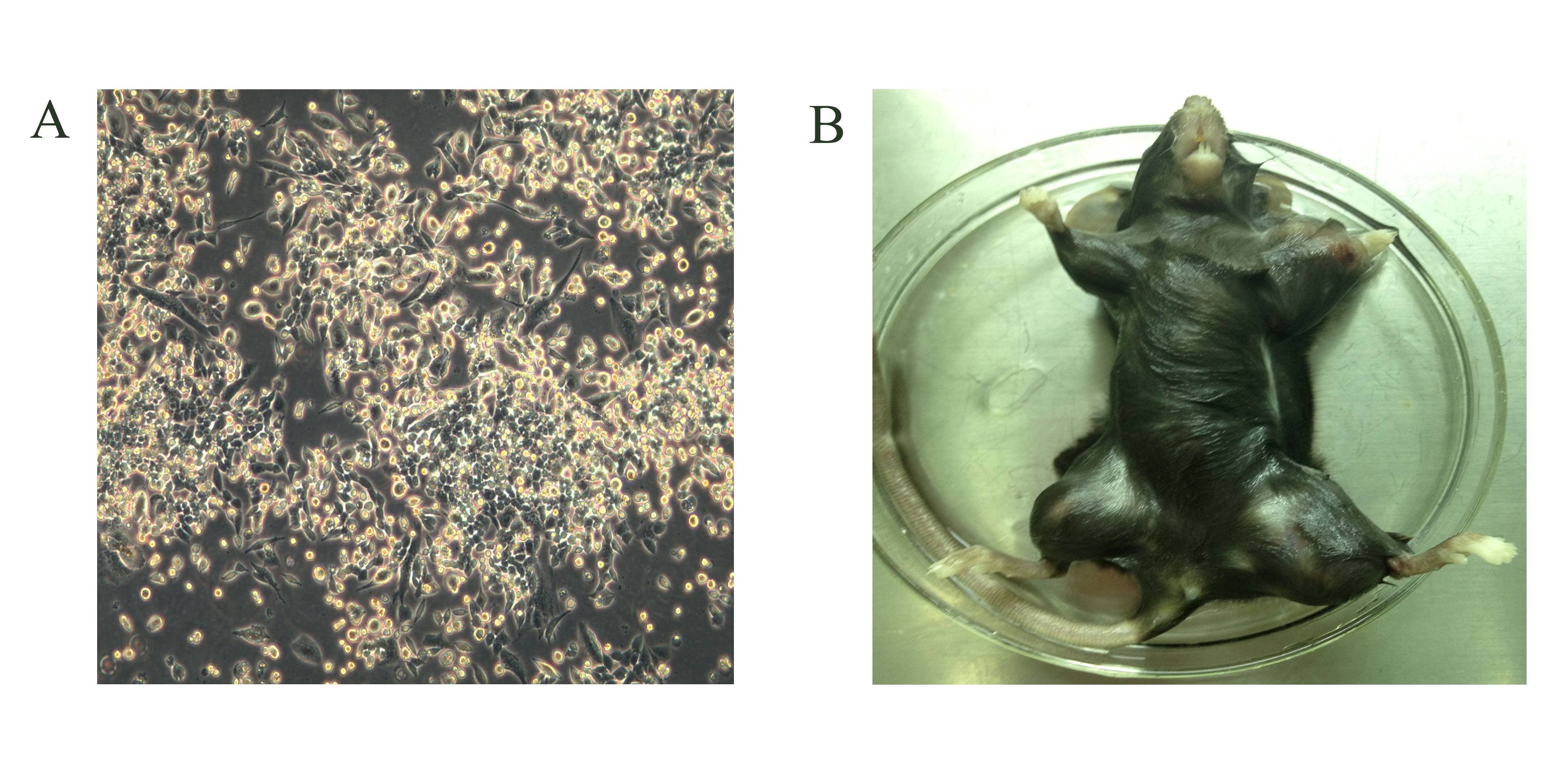

Supplement: Additional file 2: Figure S1 — Lewis lung carcinoma cells and Tumor-burdened mice. A) The Lewis lung carcinoma cells in growth logarithm period. B) Tumor-burdened C57BL/6 mice were raised for 4 weeks until the lump grew up to 3 × 3 cm. [file 1479-5876-11-71-S2.tiff]

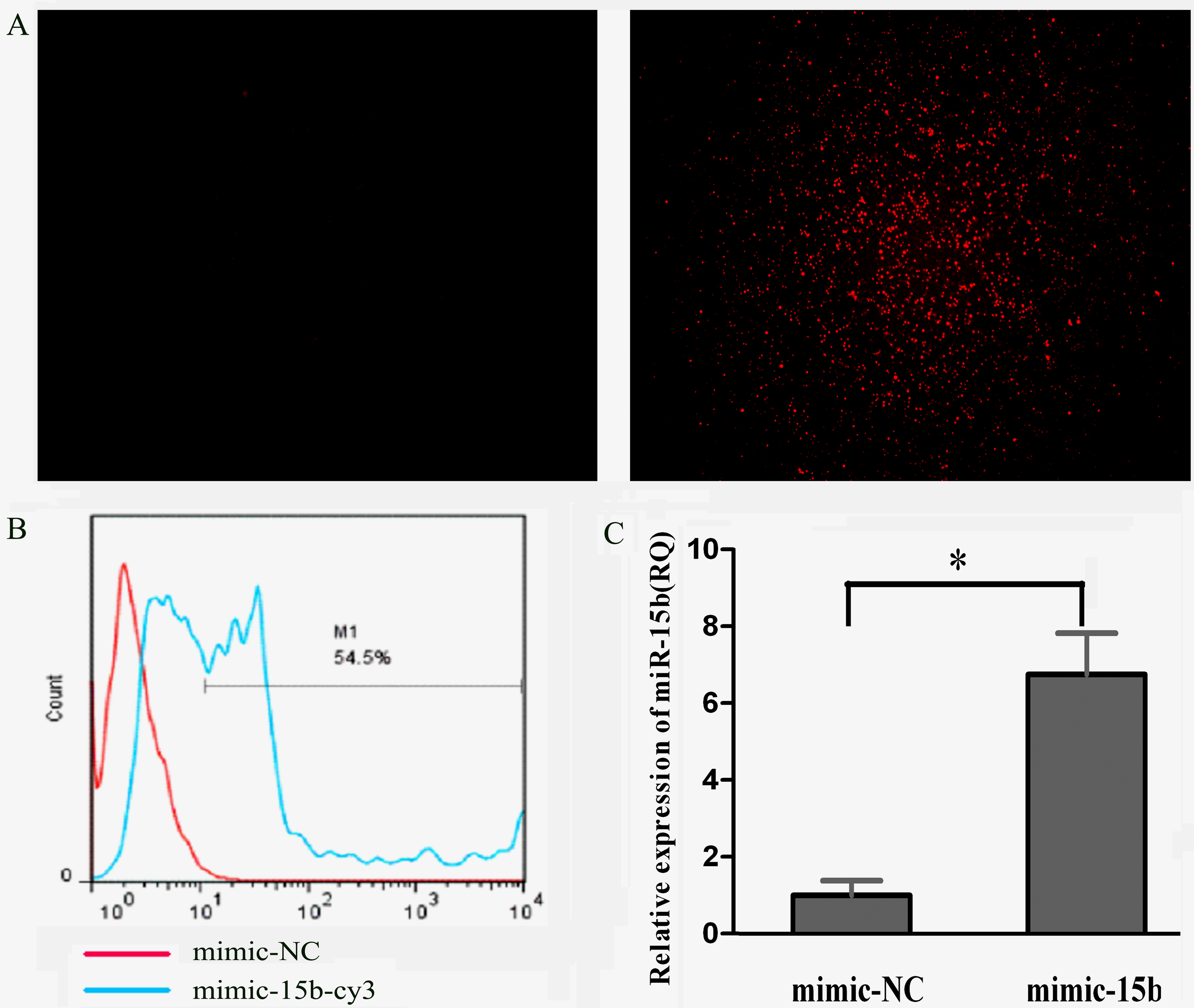

Supplement: Additional file 3: Figure S2 — Transfection of mimic-miRNA in CD8+ T cells. A) CD8+ T cells transfected with mimic-NC (left) or mimic-15b-cy3 (right) were imaged by fluorescence microscopy (red sparkle). B) Representative cytometrical plots showed transfection efficiency of mimic in CD8+ T cells. Red line represented that T cells were transfected with mimic-NC, blue line represented that T cells were transfected with mimic-15b-cy3. C) The relative expression of miRNA-15b was determined by qRT-PCR in T cells with mimic-NC or mimic-15b. U6 was used as an internal control to normalize relative amounts of miRNA. [file 1479-5876-11-71-S3.tiff]
